# Supplementary material for: Experiences and challenges of people living with multiple long-term conditions in managing their care in primary care settings in Kerala, India: A qualitative study
Source: PLoS One. 2024 Jun 13;19(6):e0305430. doi: 10.1371/journal.pone.0305430 (PMC11175503; doi:10.1371/journal.pone.0305430)
Supplement: S1 File — (DOCX) [file pone.0305430.s001.docx]

Table S1 Steps in data analysis

| Transcription | The audio recordings were transcribed into English by the research team, and one team member crosschecked the transcriptions against the audio recording for any discrepancies. |
| --- | --- |
| Familiarization | To familiarize with the data, the research team read all the transcripts thoroughly and listened to the audio recordings. |
| Coding and identifying analytical framework | Two researchers openly coded four transcribed interviews. These initial codes, and areas from the topic guide were discussed with another researcher for concordance, and the final analytical framework was developed. |
| Indexing | The developed analytical framework was used to code the complete data set (n=31). The data set was uploaded to the qualitative data software Taguette for storing and organizing the data. The coded data sets were exported to Microsoft Excel. Similar codes were grouped into categories. |
| Charting | A matrix was created by charting data, which involved summarizing the data by category with each case (participant identifier). This process facilitated the recognition of patterns in the data. |
| Mapping and interpretation | The matrices were reviewed, and connections between cases and categories were analysed. This process enabled the research team to map and interpret the collected data thematically. |

| **Participant ID** | **Age** | **Gender** | **Chronic multiple conditions** |
| --- | --- | --- | --- |
| P1 | 44 | F | Diabetes, Hypotension |
| P2 | 71 | M | Diabetes, Hypertension, COPD, Cardiovascular disease |
| P3 | 64 | M | Hypertension, Kidney disease |
| P4 | 56 | F | Diabetes, Hypertension, Cholesterol, Joint pain, COPD |
| P5 | 62 | M | Hypertension, Cardiovascular disease, Pancreatitis |
| P6 | 68 | M | Diabetes, Hypertension, Cardiovascular disease |
| P7 | 65 | F | Diabetes, Hypertension, Knee pain |
| P8 | 67 | M | Diabetes, Hypertension, Cholesterol |
| P9 | 62 | M | Diabetes, Hypertension, IVPD |
| P10 | 73 | F | Diabetes, Hypertension, Joint pain |
| P11 | 45 | F | Diabetes, Hypertension, Cholesterol, Arthritis |
| P12 | 61 | M | Diabetes, Hypertension, Cholesterol, Cardiovascular disease |
| P13 | 82 | F | Diabetes, Arthritis, Shortness of breath, airway disease |
| P14 | 62 | M | Diabetes, Hypertension, Cardiovascular disease |
| P15 | 65 | F | Diabetes, Hypertension, Cardiovascular disease, Heart valve disease, Arthritis |
| P16 | 78 | F | Diabetes, Hypertension, Cardiovascular disease, Back pain |
| P17 | 53 | M | Diabetes, Varicose vein |
| P18 | 61 | M | Diabetes, Hypertension, Chronic headache, Vertigo |
| P19 | 58 | F | Diabetes, Hypertension, Arthritis, Shoulder pain |
| P20 | 71 | F | Diabetes, Hypertension, Cholesterol, Cardiovascular disease, kidney disease |
| P21 | 65 | M | Diabetes, Hypertension, Cholesterol, Cardiovascular disease, Psoriasis |
| P22 | 62 | M | Hypertension, COPD |
| P23 | 46 | F | Diabetes, Hypertension, Cholesterol, Stroke, Thyroid, Knee pain |
| P24 | 64 | M | Diabetes, Hypertension, Cholesterol |
| P25 | 75 | M | Diabetes, Hypertension, COPD |
| P26 | 56 | F | Diabetes, Cholesterol, COPD |
| P27 | 65 | F | Diabetes, Hypertension |
| P28 | 45 | F | Diabetes, Cholesterol, Asthma, Hypothyroidism |
| P29 | 58 | F | Diabetes, Hypertension |
| P30 | 45 | M | Diabetes, Hypertension |
| P31 | 41 | M | Hypertension, Cholesterol, Fatty liver, suspected alcoholic liver issues |

Table S2 Additional demographic details of included participants

Table S3 Additional patient quotes

| Themes |  | Quotes |
| --- | --- | --- |
| **Outcomes of living with MLTCs** | a) Physical issues | I still have back pain. I have taken a lot of medicines for over the years, but still no changes. Now I do not take medicines for pain. Sometimes when it is too much I come here and show it to Sir (doctor). Sir (Doctor) also says that it is not good to have medicines for pain always. He will give Paracetamol. I cannot take most of the injections for pain; they say that I have an allergy to them. So I just go on like that(P7, 65years) |
|  |  | The main problem I am having is related to sweating of the head (Thalaneerirakkam). When it happens there will be uneasiness in the hands and the whole body, with slight pain. (P12, 61 years) |
|  |  | I do not work. I used to go to work, but now my family does not allow me to do work because of this disease (Diabetes, hypertension, and cholesterol and knee pain). I used to go for providing postnatal care and newborn care involving baby baths. So now I can't lift babies, I am very weak so I quit my job.(P11, 45 years) |
|  |  | I cannot do strenuous work as I used to do before. Recently a person called me to mulch his cow, and I couldn't do it properly, I was feeling tired.(P18, 61 years) |
|  | b)Psychological difficulties | I am having all these diseases, like sugar and all (BP, Diabetes, Cholesterol, Stroke, thyroid, knee pain. I do not cry about having all these conditions. But I am sad that I got these diseases at this younger age.(P23, 46 years) |
|  |  | In general, I will feel that I have less lifespan. I have that fear inside me. Like others, I can never walk or run freely. This kind of feeling might be there for the diabetes patients. (P24, 64 years) |
|  |  | The only tension I have is when I do not have money with me. I feel sad to depend on my children for everything. Like if I want to have tea, I should have money with me or else we will not be happy(P18, 61years) |
|  | c) challenges of self-management | I can have vegetables and all. I used to cook vegetable dishes. Thinking about income, I can't buy it all time. Food... I will eat if I can get it. If I don't get, I will eat what we usually have. If I can get I will buy and eat vegetables. If not, we will have rice with some gravy of some kind...and like that...will eat. (in a low voice) other than that...(sighs deeply)(P23, 46 years) |
|  |  | I do not use sugar; it has been a long time since I even drank tea with sugar. Then what else do I eat, some fruits that are in our house that is it. But I can't eat all fruits (P16, 78 years) |
|  |  | Actually, I cannot go for any walk; both my legs have severe pain. I have had this for a long time, around 20 years. I have not been able to pray ("niskaram"); I usually sit on a stool and do it. If I try to walk for some time or climb steps it will pain. I have actually shown it to different doctors and have had medicines for a long time, but now I don't take anything for it. I am allergic to most pain medicines (P7, 65 years) |
|  |  | They (Doctors) advised me to walk daily. I have issues with varicose veins, so it is difficult to walk also. I have knee pain so I can't take the stairs, it's painful. (P11, 45 years) |
|  |  | I came today as I had breathing difficulty and had tiredness while walking. As I have palpitation, I am unable to walk. I have to stop a while in between to move forward. And I also feel it difficult to talk continuously. (P2, 71 years) |
| **Care-coordination maze** | a)Fragmentation and poor continuity of care | Then there is difficulty to reach this centre. We take a taxi or auto each time (P10, 73 years) |
|  |  | Initially I was consulting Dr X, he is a specialist. Now I am consulting Dr Y. She (Dr Y) gave me medicine for blood pressure but that caused a side effect and I developed rashes and oedema. Because of this, I consulted Dr X again and he told me to discontinue it and gave me another medicine. (P28, 45 years) |
|  |  | It was 16 years back. I had a severe cough and breathing difficulty, I had vomiting because of the cough. Then I had a sore throat. For that, I took treatment from many hospitals, admitted several times. But the cough was not relieved even after these treatments. Then last I visited a private hospital. They told me to do an ECG, blood tests, and sputum examination. After seeing the result, they gave me some medicines. And they also told me that, due to the narrowing of the airway, I might have problems with speech and watering of the eyes.(P13, 82 years) |
|  |  | I have pain in both of my legs. I have pain in my knees and in the foot and I find it difficult to touch my foot to the ground. For this, I am seeing an ortho at K (place name). Which hospital in K (place name), is it private or government? It's X (hospital) in K (place name) and it is a private hospital. If the doctor is not there I will go to Y (hospital) in P (place name). If I don't get the cheetu here, I visit that X (hospital) for pain. (P23, 46 years) |
|  | b)Medication management; an uphill battle | For few days, I have not taken any tablets and suddenly I felt weakness, slight pain and tiredness. I was taken to health (FHC) and they said ECG should be taken and it showed slight variation. Then they asked to be admitted in medical college. After that the follow-up was not done; speaking the truth. (P31, 41 years) |
|  |  | Now along with checking diabetes, BP is also checked every month. Not even a month goes by without looking at it, every month I go and check. Today also I checked and they told me it has lowered. So it is good and will continue like this by taking medicine. (P27, 65 years) |
|  |  | I have a machine at home to check my BP. I check my sugar at home in case it is high, I go to my doctor for a consultation. (P26, 56 years) |
|  |  | Yes, I take it (tablet) on time and do it (medicine management) myself. I read the name of the medicine, and I am familiar with that. Although there are 9 medicines on the prescription sheet, 2 are not needed (He showed the two medicines marked "X" on the prescription sheet). Only 7 are needed. All these I know, like the ones that need to be taken in the morning, in the afternoon, in the night likewise. (P2, 71 years) |
|  |  | Interviewer: Which tablet is taken for BP? Respondent: I do not know its name. It is a small orange colour tablet. Interviewer: Do you identify your medicines by its colour? Respondent: Yes, I usually get the same tablet. (P30, 45 years) |
|  | c)Primary care falling short | I often think about that but here (FHC) there is no doctor like that. That's why we are going there, and there is no ortho (orthopaedic) doctor here. The doctor gives medicines for sugar and pressure from here. We can buy it from here. But we don't have doctors (specialists) for other conditions. (P23, 46 years) |
|  |  | Interviewer: Do you have someone in the FHC who listen to your health problems and manage your disease? Respondent: We cannot expect all those things; they are doing a big service by providing free treatment and medicines. What more can we expect? (P25, 75 years) |
|  |  | I never talked about this to anyone (HCPs) here. Whom will I talk to? If I talk to doctors, how can they help me? They can only give me medicines. (P24, 64 years) |
|  |  | But sometimes we may have to buy medicines from outside pharmacy. Sometimes even the medicines for sugar and pressure will not be there (in FHC) sometimes it may be missing (out of stock) for a week so we will buy from outside and next time we come here medicines would be here, so then we will again continue taking medicines from here(P7, 65 years) |
|  |  | It is difficult we have only limited time to talk with doctors. Lot of patients will be waiting outside, so they cannot spend enough time with us. They can do only that much. There are a lot of sick patients waiting outside and their condition will be more severe than ours. (P1, 44 years) |

Box S1: Topic guide

Topic guide

- Could you please tell me about the different health conditions you suffer from?

*Probes*

Could you describe the process of how these conditions were diagnosed?

Why did you seek help initially?

(Use of another healthcare facility for treatment of the same condition and reasons.)

- What does it mean to you to have several chronic diseases?

*Probes*

Could you please tell me how you felt on being diagnosed with several conditions?

Can you describe a day in your life after having to manage several conditions?

- In your opinion, what are some of the limitations that arise from your several chronic conditions (if any)?
- Could you tell me about caring for yourself with your multiple conditions?

*Probes*

How do you manage your health care appointments? (Frequency and nature of the visit to a healthcare provider/s, how do you carry information (medication/past health information) from one provider to another each provider?

How do you currently manage your medicines?

How do you engage in self-management activities- diet, physical activity, sleep habits, stress, monitoring at home/follow-up

- How do you feel about the current management of your conditions?

*Probes*

In your opinion, what are the challenging aspects of managing multiple health conditions?

Could you please explain which is your most difficult condition (in terms of your effort/difficulty) to manage and why?

- In your opinion, what would be the most critical area in your care that you need support with particularly at FHCs?

*Probes*

Reasons, possible solutions

- In your opinion, what are the different ways in which care could be improved for people with multiple health conditions?

|  | **Standards for Reporting Qualitative Research (SRQR)*** |  |
| --- | --- | --- |
|  | <http://www.equator-network.org/reporting-guidelines/srqr/> |  |
|  |  | **Page no(s).** |
| **Title and abstract** | |  |
|  | **Title** - Concise description of the nature and topic of the study Identifying the study as qualitative or indicating the approach (e.g., ethnography, grounded theory) or data collection methods (e.g., interview, focus group) is recommended | 1 |
|  | **Abstract** - Summary of key elements of the study using the abstract format of the intended publication; typically includes background, purpose, methods, results, and conclusions | 3-4 |
|  |  |  |
| **Introduction** | |  |
|  | **Problem formulation** - Description and significance of the problem/phenomenon studied; review of relevant theory and empirical work; problem statement | 5-6 |
|  | **Purpose or research questio**n - Purpose of the study and specific objectives or questions | 6 |
|  |  |  |
| **Methods** | |  |
|  | **Qualitative approach and research paradigm** - Qualitative approach (e.g., ethnography, grounded theory, case study, phenomenology, narrative research) and guiding theory if appropriate; identifying the research paradigm (e.g., postpositivist, constructivist/ interpretivist) is also recommended; rationale** | 6 |
|  | **Researcher characteristics and reflexivity** - Researchers’ characteristics that may influence the research, including personal attributes, qualifications/experience, relationship with participants, assumptions, and/or presuppositions; potential or actual interaction between researchers’ characteristics and the research questions, approach, methods, results, and/or transferability | 7 |
|  | **Context** - Setting/site and salient contextual factors; rationale** | 6 |
|  | **Sampling strategy** - How and why research participants, documents, or events were selected; criteria for deciding when no further sampling was necessary (e.g., sampling saturation); rationale** | 7 |
|  | **Ethical issues pertaining to human subjects** - Documentation of approval by an appropriate ethics review board and participant consent, or explanation for lack thereof; other confidentiality and data security issues | 9 |
|  | **Data collection methods** - Types of data collected; details of data collection procedures including (as appropriate) start and stop dates of data collection and analysis, iterative process, triangulation of sources/methods, and modification of procedures in response to evolving study findings; rationale** | 7-8 |
|  | **Data collection instruments and technologies** - Description of instruments (e.g., interview guides, questionnaires) and devices (e.g., audio recorders) used for data collection; if/how the instrument(s) changed over the course of the study | 7 |
|  | **Units of study** - Number and relevant characteristics of participants, documents, or events included in the study; level of participation (could be reported in results) | 9 |
|  | **Data processing** - Methods for processing data prior to and during analysis, including transcription, data entry, data management and security, verification of data integrity, data coding, and anonymization/de-identification of excerpts | 8 |
|  | **Data analysis** - Process by which inferences, themes, etc., were identified and developed, including the researchers involved in data analysis; usually references a specific paradigm or approach; rationale** | 8 |
|  | **Techniques to enhance trustworthiness** - Techniques to enhance trustworthiness and credibility of data analysis (e.g., member checking, audit trail, triangulation); rationale** | 8 |
|  |  |  |
| **Results/findings** | |  |
|  | **Synthesis and interpretation** - Main findings (e.g., interpretations, inferences, and themes); might include development of a theory or model, or integration with prior research or theory | 12-21 |
|  | **Links to empirical data** - Evidence (e.g., quotes, field notes, text excerpts, photographs) to substantiate analytic findings | 12-21, Online supplement Table S3 |
|  |  |  |
| **Discussion** | |  |
|  | **Integration with prior work, implications, transferability, and contribution(s) to the field -** Short summary of main findings; explanation of how findings and conclusions connect to, support, elaborate on, or challenge conclusions of earlier scholarship; discussion of scope of application/generalizability; identification of unique contribution(s) to scholarship in a discipline or field | 22-26 |
|  | **Limitations** - Trustworthiness and limitations of findings | 27 |
|  |  |  |
| **Other** | |  |
|  | **Conflicts of interest** - Potential sources of influence or perceived influence on study conduct and conclusions; how these were managed | Title page |
|  | **Funding** - Sources of funding and other support; role of funders in data collection, interpretation, and reporting | Title page |
|  |  |  |
|  | *The authors created the SRQR by searching the literature to identify guidelines, reporting standards, and critical appraisal criteria for qualitative research; reviewing the reference lists of retrieved sources; and contacting experts to gain feedback. The SRQR aims to improve the transparency of all aspects of qualitative research by providing clear standards for reporting qualitative research. |  |
|  |  |  |
|  | **The rationale should briefly discuss the justification for choosing that theory, approach, method, or technique rather than other options available, the assumptions and limitations implicit in those choices, and how those choices influence study conclusions and transferability. As appropriate, the rationale for several items might be discussed together. |  |
|  |  |  |
|  | **Reference:** |  |
|  | O'Brien BC, Harris IB, Beckman TJ, Reed DA, Cook DA.**Standards for reporting qualitative research: a synthesis of recommendations.** *Academic Medicine*, Vol. 89, No. 9 / Sept 2014  DOI: 10.1097/ACM.0000000000000388 |  |
|  |  |  |
|  |  |  |
