# Supplementary material for: Experiences and challenges of people living with multiple long-term conditions in managing their care in primary care settings in Kerala, India: A qualitative study
Source: PLoS One. 2024 Jun 13;19(6):e0305430. doi: 10.1371/journal.pone.0305430 (PMC11175503; doi:10.1371/journal.pone.0305430)
Supplement: Attachment — Submitted filename: Response to additional comments from editor.docx [file pone.0305430.s003.docx]

**Authors’ response**

We would like to thank the editor for a careful and thorough reading of our manuscript titled “Experiences and challenges of people living with multiple long-term conditions in managing their care in primary care settings in Kerala, India: A qualitative study.” Our point-by-point responses for comments are presented in italics. The page numbers and line numbers referred in this document are based on the tracked change version of the manuscript.

Sincerely,

On behalf of authors

Linju Joseph

**Additional Editor Comments:**

Dear Authors,
Thank you for carrying out the revision. The manuscript is now well structured. However, I have some points to raise before I can make a decision on your submission.

1. In table 1, there is no need to provide the initials of authors. You can consider moving the detailed table as supplementary file and can provide only the information without authors initials.

*Response*

*Thank you for your suggestion. We have now moved Table 1 to online supplement and have removed author’s initials based on your suggestion. Page number 8, line numbers 175-176; Online supplement page number 1.*

2. I feel that you should also provide the title to figure 1 within the figure itself. As of now, the title is there as a text in the manuscript. Delete that title text from the manuscript and include that within the figure.

*Response*

*Thank you for your suggestion. We have now deleted the figure title from the manuscript and have incorporated it in the figure itself.*
